# Editorial: Hepatocellular carcinoma: from diagnostic approaches to surgical and systemic therapies

**DOI:** 10.3389/fmed.2025.1633587

**Published:** 2025-06-26

**Authors:** Pradeep Kumar Shukla, Marcello Dallio, Liliana Chemello

**Affiliations:** ^1^Department of Pathology, St. Jude Children's Research Hospital, Memphis, TN, United States; ^2^Department of Precision Medicine, Hepatology and Digestive Endoscopy, University of Campania “Luigi Vanvitelli, ” Naples, Italy; ^3^Department of Medicine-DIMED, University-Hospital of Padova, Padua, Italy

**Keywords:** HCC, primary liver cancer, cirrhosis, radiomics, liver diseases

Hepatocellular carcinoma (HCC) represents a significant global health challenge, being one of the most common primary liver tumors worldwide. Its complex pathogenesis and often late presentation pose significant obstacles to effective management. The variability of HCC burden, its biological behavior, and often the underlying liver dysfunction require a multifaceted research approach, spanning computational modeling, biomarker identification, and novel therapeutic strategies, which are the emerging trends and future prospects in this rapidly evolving field. This Research Topic offers a window into the latest efforts to improve the clinical management of HCC patients by combining data science, interventional medicine, and molecular oncology.

Recent research in HCC has provided valuable insights into biomarkers and the role of inflammation in disease progression, paving the way for improving diagnostic tools and prediction algorithms. Li et al. constructed a prognostic model for HCC based on inflammation-related genes. This bioinformatics-based approach offers a promising tool for predicting patient outcomes and personalizing treatment strategies, underlining the important role of inflammation in HCC progression and the value of integrating molecular data into clinical practice. Equally important is the integration of biologically meaningful clinical variables into predictive frameworks. The platelet count to spleen diameter ratio (PSL), a non-invasive surrogate for portal hypertension, has emerged as a powerful predictor of survival in cirrhosis-associated HCC as presented by Yan et al. Its incorporation into LASSO-derived nomograms has yielded models that surpass traditional tools in discriminative power and calibration. This approach underscores a growing recognition that variables reflecting the liver's functional status and hemodynamics are as crucial to prognosis as tumor-specific metrics. A new recent terminology proposed for definition of fatty liver disease or “metabolic disfunction-associated steatotic liver disease (MASLD)” was introduced being close associated with presence of cardiometabolic indexes. Therefore, the cross-sectional study by Bai et al., who explored the link between albumin-corrected anion gap (ACAG), which reflects the body's acid-base balance, and hepatic metabolic syndrome using 2017–2018 NHANES data, reveals a positive correlation between elevated ACAG and—NAFLD or MASLD—, positioning ACAG as a potential early biomarker for metabolic liver disease. Further research is needed to confirm these findings and explore the underlying mechanisms, laying the foundation for new diagnostic strategies in MASLD, which affects approximately 30% of the adult population worldwide and is closely linked to the risk of HCC. At the molecular level, a deeper understanding of HCC biology is beginning to clarify why certain patients progress more aggressively or respond differently to therapy. The protein KEAP1, a regulator of oxidative stress response, has been identified in the study of Wei et al. as a marker of poor prognosis, associated with immune dysregulation and altered liver function. High KEAP1 expression correlates with shifts in immune infiltration—specifically increased Th2 cell presence and diminished cytotoxic cell activity—suggesting a role in immune evasion. Such molecular insights not only deepen our biological understanding of HCC, but also suggest new avenues for targeted therapy and immunomodulation.

While systemic strategies are expanding, refinement of curative-intent local therapies remains equally critical. For early-stage HCC, particularly single tumors ≤ 5 cm, image-guided radiofrequency ablation (RFA) remains a mainstay. However, its efficacy can be compromised by incomplete ablation or recurrence. A novel approach presented by Xie et al. which combined transarterial chemoembolization (TACE) with multi-image guidance during RFA has shown promising results, achieving high technical success and long-term survival with minimal complications. Furthermore, the debate is also on the use of adjuvant systemic treatment, as already used in other cancer types, even in early HCC. A meta-analysis by Tuersun et al. evaluates the efficacy of neoadjuvant immune checkpoint inhibitors (ICIs) in cases with resectable HCC. This study shows improved pathological responses without significantly increasing adverse events, suggesting a promising new direction for perioperative cancer care, and supporting further investigation into the use of immunotherapy also in early-HCC stage management, at least for the patients with the more invasive histotypes.

Also therapeutically, the landscape is evolving. Patients with advanced HCC and high tumor burden—long considered to have limited treatment options—are now benefiting from strategic combinations of locoregional and systemic therapies. Evidence from a multicenter analysis by Zhao Z. et al. suggests that hepatic arterial infusion chemotherapy (HAIC), when combined with Lenvatinib and Tislelizumab, significantly improves both overall and progression-free survival, with manageable toxicity. This multimodal approach leverages the cytotoxic precision of HAIC with the systemic control of immunotherapy and targeted agents, offering a new avenue for patients traditionally underserved by monotherapies. A similar study in patients staged Barcelona Clinic Liver Cancer B (intermediate-stage) or unresectable HCC, proposed the TACE associated to a systemic regimen with Atezolizumab plus Bevacizumab, to offer the possibility for downstaging to meet the criteria for curative surgery and the possible restitution of patient life expectancy, as reported in the article of Ai et al.. These new multimodal approach on interventional oncology propose a greater effectiveness, by improving both the precision and duration of local treatments, particularly for advanced HCC.

However, resistance to Regorafenib in advanced HCC continues to pose challenges. A study by Hu, Shi et al. reveals that bypass activation of the epidermal growth factor receptor (EGFR) pathway mediates acquired resistance. This suggests that combination therapies targeting EGFR could restore treatment efficacy, providing new strategies to overcome drug resistance and improve patient outcomes.

In the context of hepatitis B virus-related HCC, Hu, Yang et al. also compare the effects of tenofovir (TDF) and entecavir (ETV) in patients undergoing liver resection. TDF was found to offer superior benefits in reducing recurrence and improving survival post-surgery, highlighting its potential for guiding antiviral therapy selection. Further prospective trials are needed to validate these findings and refine guidelines for HBV-HCC management.

Another important aspect certainly concerns the desirable application of shared protocols in the study of images obtained with CT/MR. The standardized technique, implemented using an appropriate tracer concentration, improves image quality to facilitate HCC reconstruction and can be decisive for studying the tumor vasculature, especially to reveal microvascular invasion in cases with advanced HCC, as demonstrated in the study of Peng et al.. This can allow us to obtain the best quality for the most accurate study of the tumor stage, avoiding additional sessions. In the article by Huang et al., also quantification of intra-tumoral fat, which is commonly detected during MRI, serves as an important ancillary feature for the diagnosis of HCC, and furthermore it allows distinguishing the low-proliferative tumor pattern with better prognostic prediction, effectively influencing the therapeutic choice.

Finally, a note of caution is reiterated in the systematic review and meta-analysis of Yan et al., which highlight the clinical relevance of the rare arc of Bühler, particularly in abdominal surgery and interventional radiology. The study emphasizes also the utility of computed tomography angiography (CTA) and digital subtraction angiography (DSA) for preoperative diagnosis, which helps reduce intraoperative risks by increasing awareness of vascular anomalies, which can produce severe complications in treated patients.

Nowadays the diagnostic techniques and therapeutic strategies, especially by integration of machine learning and artificial intelligence to clinical practice, drastically improved early detection, personalize treatment plans, and ultimately achieve better outcomes for patients with HCC. A common thread across these contributions is the pressing need for more accurate prognostic tools. As current staging systems such as AJCC-TNM fall short in capturing the true clinical diversity of HCC—particularly in advanced or multifocal disease—researchers are increasingly turning to data-driven approaches. Recent work by Shen et al. harnessing large-scale registry data has demonstrated that machine learning algorithms, especially gradient boosted models, can outperform traditional statistical methods in stratifying risk and predicting survival. These models not only show high accuracy in validation cohorts but also offer interpretability through techniques like SHAP, supporting their integration into clinical workflows. This evolution in prognostic modeling signals a shift toward personalized risk assessment in HCC, where prediction is tailored to the individual rather than to broad disease categories.

Early recurrence of HCC after surgical resection occurs in more than 50% of cases. This suggests the need to improve prediction accuracy compared to traditional methods. The radiomics-deep learning integrated model approach presented by Zhao Y. et al. showed an AUC of 0.917, with diagnostic accuracy of 88.6%, largely outperforming traditional clinical models in predicting early HCC recurrence and could significantly improve risk stratification and prognosis for HCC patients undergoing previous curative resection. A similar integrated model was also proposed in the study by Zhang M. et al., who applied a clinical selection of patient characteristics (age at DGN, TNM stage, and metastasis) and tested various machine learning algorithms, confirming enhanced power for outcomes prediction in patients treated with TACE for HCC by the model XGBoost. The knowledge and application of these models is fundamental to improve the diagnosis and prognosis in HCC cases. The gadoxetic acid-enhacement MRI for pre-operative evaluating of vessels encapsulating tumor clusters (VETC) patterns in HCC results of crucial importance, as the VECT phenotype, showing intra-tumoral blood vessels enveloped sections, exerts an impact on early recurrence and overall survival of HCC and proved higher sensitivity to angiopoietin-2 inhibitors therapy. A nomogram presented by Zhang J. et al. visualizing the integrated model, composed of selected traditional imaging features (i.e., necrosis or ischemia, wash-out and lesion-to-liver ratio) and radiomics application on arterial, portal and hepato-biliary phase, showed a significant (AUC 0.873; 95%CI 0.821–0.925) accuracy in predicting VETC patterns by preoperative MRI analysis. Thus, VECT profile may provide guidance for prevention and treatment of patients with HCC-recurrence.

**Figure 1:**
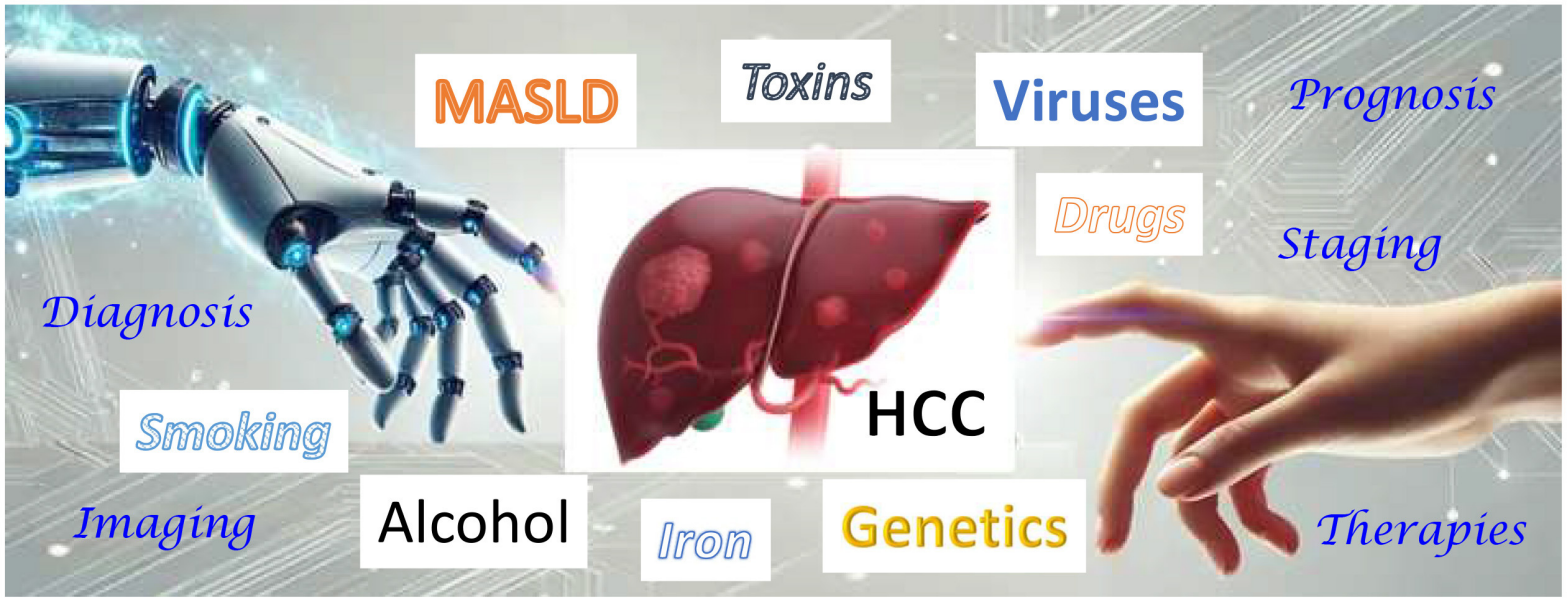
Ethiologies of hepatocellular carcinoma (HCC) and clinical interventional processes.

At the end, we must not give up, because HCC appears to be an indolent but progressive liver tumor, with a remaining patient life expectancy of 5–8 years from diagnosis. Over the last decade, local and minimally invasive treatments and complex-combined strategies of surgery and systemic immunotherapy have been tested with the aim of proposing, in the majority of cases, a curative therapy or, when possible, a liver transplant. The studies presented in Research Topic point toward these new frontiers, even if much remains to be discovered on the mechanisms of carcinogenesis, which often develop through variable forms of liver damage (Figure 1), and do not yet reveal a common biological and molecular pathway.

